# Retraction: The controlled synthesis of g-C_3_N_4_/Cd-doped ZnO nanocomposites as potential photocatalysts for the disinfection and degradation of organic pollutants under visible light irradiation

**DOI:** 10.1039/d6ra90083f

**Published:** 2026-07-30

**Authors:** Mudassar Sher, Mohsin Javed, Sammia Shahid, Shahid Iqbal, Muhammad Azam Qamar, Ali Bahadur, Muhammad Abdul Qayyum

**Affiliations:** a Department of Chemistry, School of Science, University of Management and Technology Lahore 54770 Pakistan mohsin.javed@umt.edu.pk; b Department of Chemistry, School of Natural Sciences (SNS), National University of Science and Technology (NUST) H-12 Islamabad 46000 Pakistan shahidiqbal.chem@sns.nust.edu.pk; c Department of Transdisciplinary Studies, Graduate School of Convergence Science and Technology, Seoul National University Seoul 08826 South Korea alibahadur138@snu.ac.kr; d Department of Chemistry, Division of Science and Technology, University of Education Lahore Pakistan Lahore Pakistan

## Abstract

Retraction of ‘The controlled synthesis of g-C_3_N_4_/Cd-doped ZnO nanocomposites as potential photocatalysts for the disinfection and degradation of organic pollutants under visible light irradiation’ by Mudassar Sher *et al.*, *RSC Adv.*, 2021, **11**, 2025–2039, https://doi.org/10.1039/d0ra08573a.

The Royal Society of Chemistry hereby wholly retracts this *RSC Advances* article due to concerns with the reliability of the data.

The XRD patterns for 60% g-C_3_N_4_/Cd–ZnO NCs before use, after 5-times use and after 10-times use in Fig. 12a appear identical.

The ESR data in Fig. 13b contains an unexpected repeating pattern.

The authors provided a response and new data, but they did not address the concerns, and they were not able to provide the original raw data.

Given the significance of these concerns, the findings presented in this paper are no longer reliable.

Mudassar Sher, Mohsin Javed, Sammia Shahid, Shahid Iqbal, and Muhammad Azam Qamar oppose this retraction decision. The other authors did not respond to any correspondence regarding the retraction.

Laura Fisher

16^th^ July 2026

Executive Editor, *RSC Advances*

